# DNA METHYLATION AGE ACCELERATION MEDIATES THE ASSOCIATIONS BETWEEN EARLY-LIFE CIRCUMSTANCES AND DEMENTIA RISK

**DOI:** 10.1093/geroni/igae098.4288

**Published:** 2024-12-31

**Authors:** Fan Pu, Zhuoer Lin, Jiao Yu, Zuyun Liu, Xi Chen

**Affiliations:** Yale University, New Haven, Connecticut, United States; University of Illinois Chicago, Chicago, Illinois, United States; Yale University, New Haven, Connecticut, United States; School of Medicine, Zhejiang University, Zhejiang, China (People’s Republic); Yale University, New Haven, Connecticut, United States

## Abstract

Dementia represents critical public health challenges and exerts an immense economic burden. While mounting evidence has linked early-life circumstances to dementia risk, prior research mainly focused on single factors, failing to capture the complex interplay of comprehensive early-life circumstances and the underlying biological pathways. This study aims to fill the gap by exploring how multidimensional early-life circumstances, spanning family education, relationships, financial status, regional context, and school experiences, contribute to dementia risk in later life. Particularly, we investigate the role of DNA methylation age acceleration (DNAmAgeAccel) as a strong mediator of the associations. Using data from 4,018 American adults aged 50 and older from the Health and Retirement Study, we assessed dementia using the Langa-Weir Classification of Cognitive Function and DNAmAgeAccel through the residuals of 11 DNAm clocks. Our multivariate regression analyses demonstrated significant associations between early-life circumstances and dementia risk. Notably, adverse family education environments, such as mother’s low educational attainment and absence of time/attention from mother, along with poor family relationships, were associated with an increased risk of dementia. In contrast, positive school experiences, like engagement in creative arts in high school, were protective. Importantly, DNAmAgeAccel showed significant mediation effects, especially in education-related factors. For instance, DNAmAgeAccel mediated 15.1% to 32.8% of the association between maternal education and dementia risk. These findings highlight the enduring impact of multidimensional early-life circumstances on late-life cognitive health and suggest DNAmAgeAccel as a key biological pathway linking early adversity to dementia risk.

